# Molecular Design of H_2_ Storage/Release Devices: A Direct Ab Initio MD Study

**DOI:** 10.3390/nano15191498

**Published:** 2025-10-01

**Authors:** Hiroto Tachikawa

**Affiliations:** Division of Applied Chemistry, Faculty of Engineering, Hokkaido University, Sapporo 060-8628, Japan; hiroto@eng.hokudai.ac.jp

**Keywords:** H_2_ storage, reversible device, ab initio MD, hydrogen addition, hydrogen abstraction, activation energy, reaction dynamics, reaction time

## Abstract

To advance a hydrogen-based energy society, the development of efficient hydrogen storage materials is essential. In particular, such materials are expected to be lightweight and chemically stable. Moreover, they must allow for easy storage and release of hydrogen. In this study, we theoretically designed hydrogen storage and release devices based on graphene (GR)—a lightweight and chemically stable material—using a direct ab initio molecular dynamics (AIMD) approach. The target reaction in this study is the hydrogen abstraction from hydrogenated graphene, H-(GR)-H, by hydrogen atom, resulting in molecular hydrogen formation: H-(GR)-H + H → GR-H + H_2_. Hydrogen atom (H) can be readily generated through the discharge of H_2_ gas. The calculated activation energy was −0.3 kcal/mol. The direct AIMD calculations showed that the hydrogen abstraction reaction proceeds without the activation barrier, and H_2_ is easily formed by the collision of H atom with the H-(GR)-H surface. For comparison, the addition reaction of hydrogen atom to the graphene surface was investigated: GR + H → GR–H. The activation energies were calculated to be 5–7 kcal/mol. These energetic profiles indicate that both hydrogen storage and release proceed with low and negative activation energies, respectively. On the basis of these calculations, H_2_-storage/release device was theoretically designed.

## 1. Introduction

Carbon materials are among the most promising candidates for hydrogen (H_2_) storage due to their lightweight nature and chemical stability [1,2,3]. For instance, the interactions between carbon nanotubes (CNTs) and H_2_ [4,5,6], as well as between graphene (GR) and H_2_, have been extensively studied [7,8,9]. However, the binding energies of H_2_ to these carbon materials are typically of less than 1 kcal/mol [10,11], which results in low hydrogen storage efficiency for pristine carbon materials.

To address this limitation, alkali metals such as lithium atoms or ions have been doped onto carbon surfaces. For example, lithium-doped graphene (GR-Li) has been shown to increase the adsorption energy of H_2_ to approximately 5–6 kcal/mol [12,13]. Moreover, it has been reported that up to 10 H_2_ molecules per Li atom can be efficiently bound to GR-Li. Other metals, such as Na, K, Mg, and Al, have also been shown to have hydrogen storage capacity [14,15,16,17].

In the present study, we theoretically explore the potential of hydrogen storage via chemical bonding between GR nanoflakes and hydrogen atoms. One known approach for chemical hydrogen storage is the hydrogenation of aromatic hydrocarbons (organic chemical hydride method), which allows for both the storage and transport of hydrogen in a chemically bound form. A representative example is the hydrogenation of benzene to cyclohexane [18,19,20,21]:

benzene + 3 H_2_ → C_6_H_12_ (cyclohexane)

Cyclohexane is produced by the addition of hydrogen atoms to the π-bonds of the benzene ring. This product can be transported, and hydrogen can later be released via catalytic dehydrogenation. Thus, both metal doping and chemical hydrogenation are considered viable strategies for H_2_ storage.

**Figure 1:**
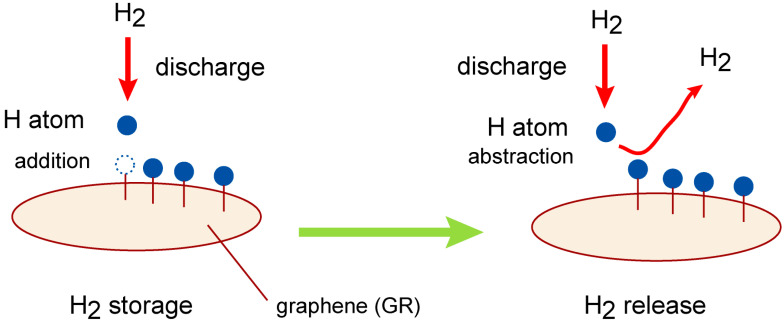
Schematic illustration of reaction model in the present study. The reactions are composed of H_2_ storage and release processes on graphene (GR) nanoflake.

In the present study, we perform a molecular-level design of a GR-based hydrogen storage system using direct ab initio molecular dynamics (AIMD) simulations. Figure 1 shows a schematic illustration of the reaction model investigated in this study. The overall process can be described as follows. Molecular hydrogen (H_2_) is readily dissociated into atomic hydrogen via electrical discharge.

H_2_ + hν (discharge) → H + H

The resulting hydrogen atoms are then introduced to the graphene surface, where they react with carbon atoms to form C–H bonds on GR surface:

GR + H → GR–H (hydrogen atom addition)

GR–H + H → H–(GR)–H (H_2_ storage)

Through this process, H_2_ is stored on GR surface in the form of two hydrogen atoms (H_2_ storage). The release of hydrogen can occur via the following reactions:

H–GR–H + H → GR–H + H_2_ (H_2_ release)

GR–H + H → GR + H_2_

The H atom, generated by discharge of H_2_, attacks the C-H hydrogen of H-(GR)-H, and then H_2_ is formed (H_2_ molecule). The primary reaction of interest in this study is the hydrogen release process (H_2_ release):

H–(GR)–H + H → GR–H + H_2_ (R1)

For comparison, we also investigate the energetics of the hydrogen addition reaction:

GR + H → GR–H(R2)

Both the energetic and the reaction times for hydrogen storage and release are evaluated using direct AIMD simulations.

For Reaction (2) [22,23], we previously investigated the activation and reaction energies by means of density functional theory (DFT) methods. Also, direct H_2_ addition reaction to GR:

H_2_ + GR + H → H-(GR)-H (R3)

was investigated [24]. The activation energies of Reactions (2) and (3) were calculated to be 5–7 and 80 kcal/mol, respectively [22,23,24]. These results indicated that the direct H_2_ addition to GR (Reaction (3)) is impossible under the normal condition. In contrast, Reaction (2) is possible in the normal condition if H atom is generated.

## 2. Computational Details

### 2.1. Ab Initio Calculations

**Figure 2:**
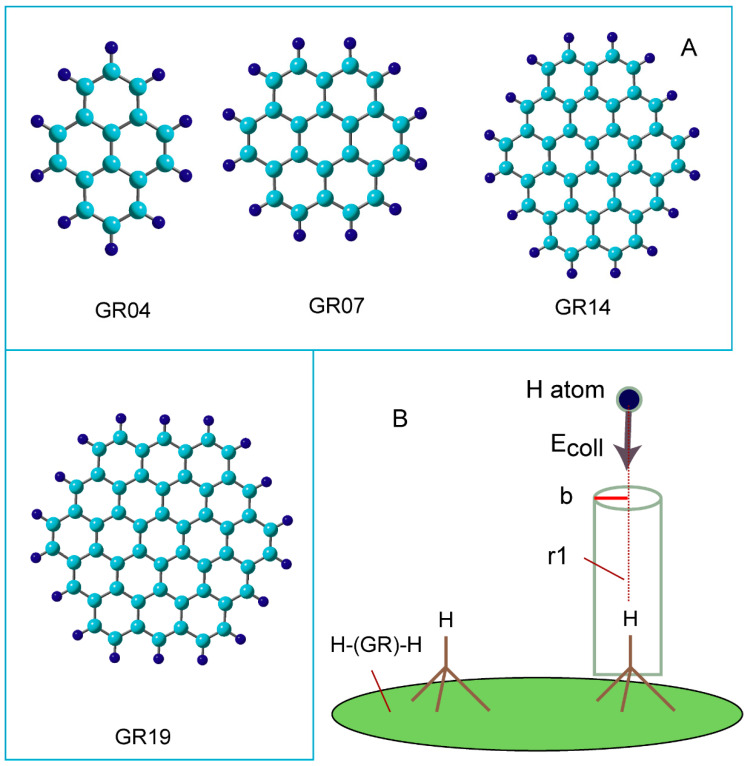
(**A**) Graphene (GR) nanoflakes used in the present study as a carbon material. GR04, GR07, GR14, and GR19 are composed of 4, 7, 14, and 19 benzene rings, respectively. The carbon atoms in the edge region are terminated by hydrogen atoms. (**B**) Initial geometry configuration in hydrogen abstraction reaction, H + H-(GR)-H → H_2_ + (GR)-H, at time zero in direct ab initio MD calculations. Notation “*r*1” means distance of collisional hydrogen atom from hydrogen atom on H-(GR)-H (hydrogenated GR), and “b” means the impact parameter in collision system.

Four types of graphene nanoflake composed of 4, 7, 14, and 19 benzene rings were used in this study, and are referred to as “GR04, GR07, GR14, and GR19” hereafter, respectively. Figure 2A shows the structures of GRs used in this calculation. DFT calculations were performed using the CAM-B3LYP Coulomb-attenuating exchange–correlation energy functional [25] with the 6-31G(d) and 6-311G(d,p) basis sets [26]. The structure of GRs was first optimized, after which one or two hydrogen atoms were placed in the central region of the GR. The mono- and di-hydrogenated GRs were expressed as GR-H and H-(GR)-H, respectively. The structures of GR-H and H-(GR)-H were then fully optimized. The binding energy of the H atom to GR is defined as follows:

(1) $$-E_{bind}=E\left(GR-H\right)-[E\left(H\right)+E\left(GR\right)]$$

where *E(X)* is the total energy of *X*. The *H* atom binds exothermally to the *GR* when *E_bind_(H)* is positive.

Atomic charges were calculated using the natural bond population analysis (NPA) algorithm [27]. All calculations were performed using the Gaussian 09 software package [28]. We previously investigated interactions between graphene and various molecules using DFT calculations at the same level of theory [13,17,29,30]. A similar technique was used for the H-(GR)-H system in this study.

### 2.2. Direct AIMD Calculations

Figure 2B shows the initial configuration of the collision reaction system. For the direct AIMD calculations, the H-(GR)-H cluster was optimized at the CAM-B3LYP/6-31G(d) level. Subsequently, a H atom was positioned above the cluster at a distance of r1 = 4.0–4.5 Å; the trajectory began from this position. The collision energies of H atom (E_coll_) were set to 1–15 kcal/mol at time zero. A near collinear collision was assumed: the impact parameter (*b*) was *b* = 0–0.5 Å. The excess energy, momentum vector, and rotational temperature of the reaction system were assumed to be zero (at 0 fs). The equations of motion for *N* atoms in the reaction system are given by:

(2) $$\begin{array}{l} \frac{dQ_{j}}{dt}=\frac{\partial H}{\partial P_{j}}\\ \frac{dP_{j}}{dt}=-\frac{\partial H}{\partial Q_{j}}=-\frac{\partial U}{\partial Q_{j}} \end{array}$$

where *j* = 1–3 *N*, *H* is the classical Hamiltonian and *Q_j_* is the Cartesian coordinate of the *j*-th mode, *U* is the potential energy, and *P_j_* is the conjugated momentum; these equations were numerically solved (NVE ensemble). The velocity Verlet algorithm, with a time step of 0.10–0.25 fs, was used to solve the equations of motion for the system. The maximum simulation time was 2.0 ps. The total energy drift in all trajectory calculations was <0.01 kcal/mol. The numbers of trajectory runs were 22 (E_coll_ = 1.0), and 5 (E_coll_ = 2.0–15), respectively. The effects of the functional on the reaction mechanism were investigated using the APFD functional and compared with those of the CAM-B3LYP functional. Direct AIMD calculations were performed using custom-made AIMD codes [31,32,33,34].

## 3. Results

### 3.1. Structures at Stationary Points for the Hydrogen Abstraction Reaction

**Figure 3:**
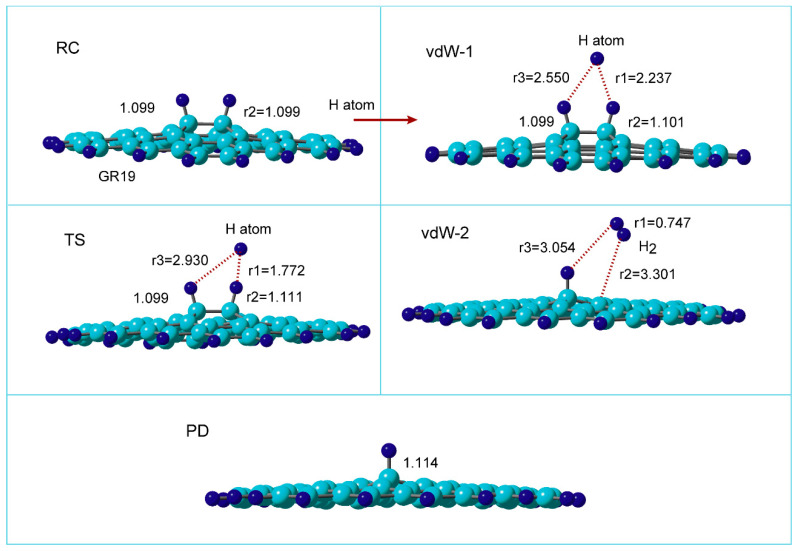
Optimized structures of reaction system along the hydrogen abstraction reaction: hydrogenated GR at reactant state (RC: H-(GR)-H), van der Waals states (vdW-1 and 2), transition state (TS), and product state (PD: GR-H). The distances and bond lengths are in Å. The calculations were carried out at the CAM-B3LYP/6-311G(d,p) level. GR19 was used as the GR nanoflake.

Figure 3 shows the optimized structures along the reaction coordinate for the reaction:

H-(GR)-H + H → H-(GR) + H_2_

where H-(GR)-H means hydrogenated GR (two H atoms are added to the surface of GR).

At reactant state (RC), the C-H bond length was calculated to be r2 = 1.099 Å. After the injection of hydrogen (H) atom to H-(GR)-H, the van der Waals (vdW) complex was first formed on the surface in the initial state of the reaction (denoted to vdW-1). The H atom was located between two surface C-H sites and made the bridge structure composed of C-H--H--H-C (r1 = 2.237 and r3 = 2.550 Å). The C-H bond was slightly elongated by the interaction with the H atom (the C-H bond length was changed from r2 = 1.099 to 1.101 Å).

Next, the reaction reached the transition state (TS) via vdW-1. The H atom was located at r1 = 1.772, and r3 = 2.930 Å at TS. The C-H bond of the surface C-H site was elongated at TS (r2 = 1.111 Å). The H-H bond (r1 = 1.772 Å) was significantly longer than that of H_2_ molecule in gas phase (r(H-H) = 0.746 Å). After TS, a H_2_ molecule was formed by H atom abstraction.

In the final state of the reaction, a vdW complex was formed (vdW-2), where H_2_ was weakly bound to the radical site of the surface. H_2_ was located at r2 = 3.301 Å from the surface. The H-H bond of H_2_ was r1 = 0.747 Å, which is a normal bond length of H_2_ molecule. After the H_2_ reduction from H-(GR)-H, the product (PD) remained as H-(GR).

### 3.2. Energy Diagram for the Hydrogen Abstraction Reaction

**Figure 4:**
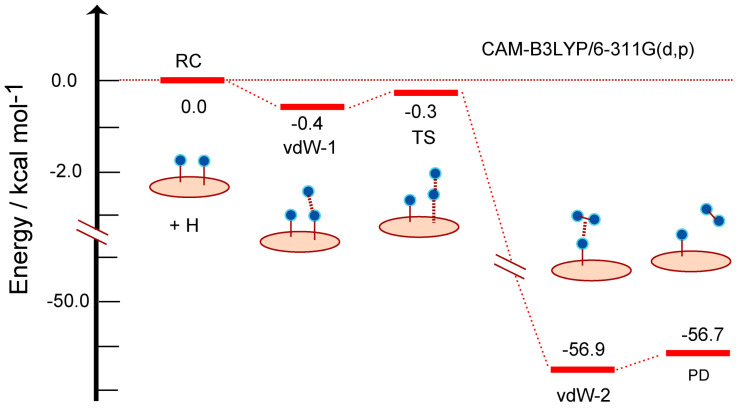
Energy diagram of reaction system along the hydrogen abstraction reaction: hydrogenated GR14 at reactant state (RC: H-(GR)-H), van der Waals states (vdW-1 and 2), transition state (TS), and product state (PD: GR-H). The distances and bond lengths are in Å. The calculations were carried out at the CAM-B3LYP/6-311G(d,p) level. GR19 was used as GR nanoflake.

The energy diagram for the reaction is given in Figure 4. The energy level of RC corresponds to the total energy of H-GR-H + H. The initial vdW state (vdW-1) was −0.4 kcal/mol lower in energy than that of RC (zero level). The energy of TS was −0.3 kcal/mol, indicating that the hydrogen abstraction reaction can proceed without activation energy.

After TS of H abstraction, the energy of reaction system decreased largely to −53.1 kcal/mol because the H-H bond was newly formed by the formation of H_2_. The energy level of vdW state at the PD state was −53.1 kcal/mol (vdW-2). The energy of the product state (PD) (−52.9 kcal/mol) was slightly higher than that of vdW-2, where H_2_ was dissociated from the surface. The reaction can be expressed as follows:

RC[H(radical) + H-(GR)-H] → vdW-1 → TS → vdW-2 → PD[(GR)-H + H_2_]

This reaction spontaneously occurs without activation barrier.

### 3.3. Intrinsic Reaction Coordinate

The intrinsic reaction coordinate (IRC) for the hydrogen abstraction reaction from H-(GR)-H was calculated at the CAM-B3LYP/6-31G(d) level. The result is given in Figure S1 (in Supporting Information: SI). The point at *s* = 0.0 in (amu)^1/2^ Bohr corresponds to TS structure in the reaction, and the points at *s* = −0.5 and *s* = 3.0 indicate the reactant and product regions, respectively. The shape of IRC suggested that the energy decreases drastically after the TS point because a H_2_ molecule with a strong H-H bond is formed. The structure at *s* = −0.5 was close to that of vdW-1. After TS, the energy decreased largely to −23.0 kcal/mol (*s* = 1.2) and −55.0 kcal/mol (*s* =3.2). At *s* = 3.2, the H_2_ molecule dissociated from H-GR as a H_2_ molecule. This IRC calculation indicated that the TS structure connected between RC and PD states for the H abstraction reaction.

### 3.4. Reaction Dynamics

**Figure 5:**
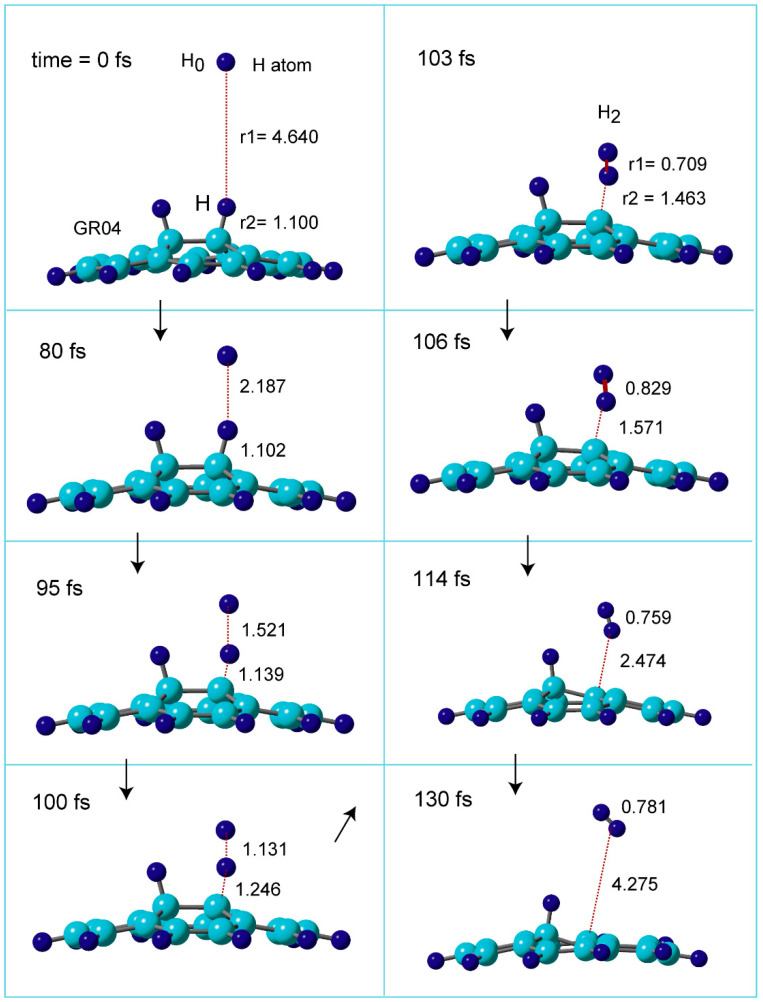
Time evolution of snapshots of the reaction system for hydrogen abstraction reaction, H + H-(GR)-H → H_2_ + GR-H. Direct AIMD calculation was performed at the CAM-B3LYP/6-31G(d) level. The CAM-B3LYP/6-31G(d)-optimized geometry of H-(GR)-H was used as the initial geometry at time zero. The distances and bond lengths are in Å. GR04 was used as the GR nanoflake.

The direct AIMD calculations were carried out at the CAM/6-31G(d) level of theory. GR04 was used as the graphene nanoflake. The collision energy was E_coll_ = 1.0 kcal/mol. The snapshots of hydrogen abstraction reaction on GR are illustrated in Figure 5, where the reaction is expressed as H + H-(GR)-H → H-(GR) + H_2_.

In the initial structure at time = 0.0 fs, the H atom was located at r1 = 4.640 Å. The C-H distance of surface as r2 = 1.100 Å. At time = 80 fs, the H atom was located at r1 = 2.187 Å, and the C-H bond was r2 = 1.102 Å. The H atom was closer to the C-H (r1 = 1.521 and r2 = 1.139 Å) at 95 fs. At 100 fs, the structure was close to that of TS (r1 = 1.131 and r2 = 1.246 Å), indicating that the C-H bond was largely elongated by the H abstraction (1.246 Å). At 103 fs, H_2_ molecule was completely formed: H-H bond length was r1 = 0.709 Å and H_2_ was located at r2 = 1.463 Å from the surface. The distances of H_2_ from the surface were changed to 1.571 Å (106 fs), 2.474 Å (114 fs), 4.275 Å (130 fs), suggesting that H_2_ leaved rapidly from the surface. Thus, the H abstraction and H_2_ formation reactions were completed at 130 fs.

**Figure 6:**
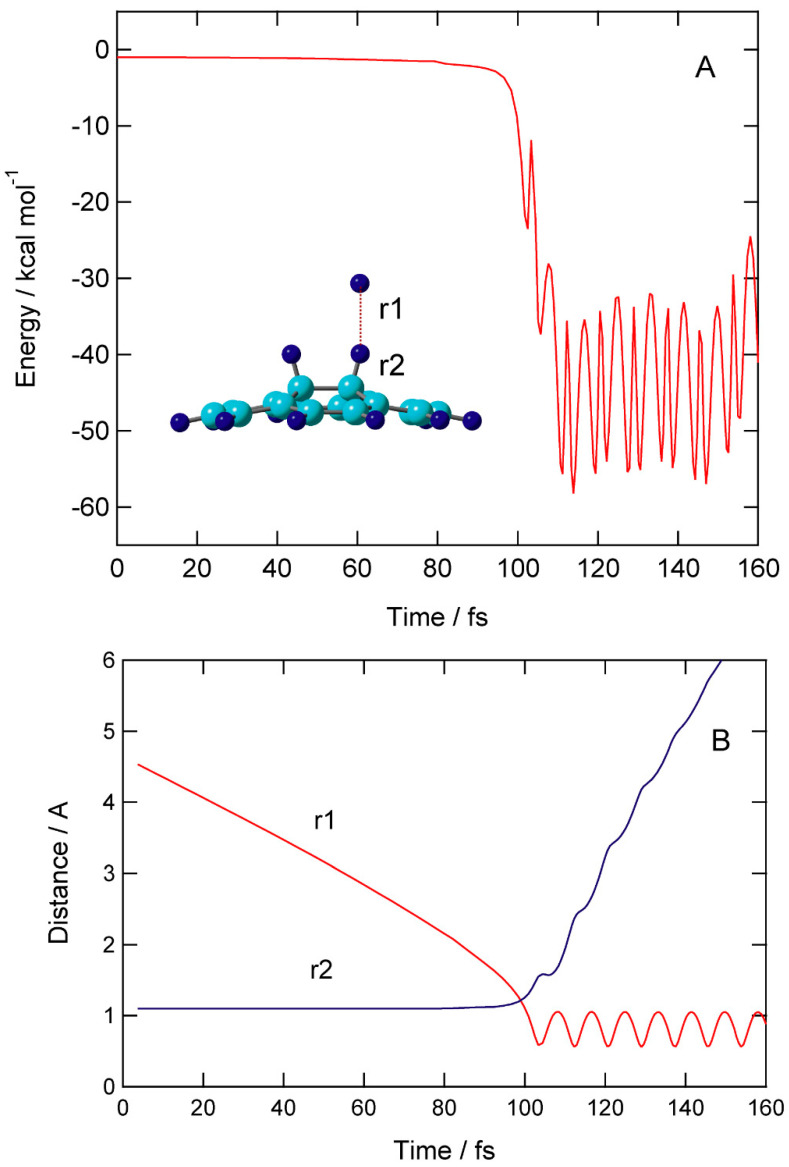
Time evolution of (**A**) potential energy and (**B**) interatomic distances of reaction system (r1 and r2). Direct AIMD calculations were performed at the CAM-B3LYP/6-31G(d) level. The CAM-B3LYP/6-31G(d)-optimized geometry of H-(GR)-H was used as the initial geometries of H-(GR)-H at time zero.

Time evolution of potential energy (PE) and interatomic distances (r1 and r2) are given in Figure 6.

At time zero, the H atom started from r1 = 4.640 Å above the surface of H-(GR)-H. The H atom gradually approached C-H site of H-(GR)-H: distances (r1, r2)= (3.460, 1.100) at 40 fs, (2.827, 1.101) at 60 fs, and (2.187, 1.102) at 80 fs. PE was slightly decreased: −0.5 kcal/mol at 80 fs. PE drastically changed around time = 100 fs, where hydrogen abstraction rapidly occurred a time 95–105 fs. The H-H bond was newly formed after abstraction, and the H_2_ molecule was formed at this time. After H_2_ formation, H_2_ was rapidly dissociated from the surface: the distances of H_2_ from the surface were 3.2 Å (120 fs) and 5.0 Å (140 fs). PE vibrated largely in the range of (−40)–(−55) kcal/mol after the H_2_ dissociation.

**Figure 7:**
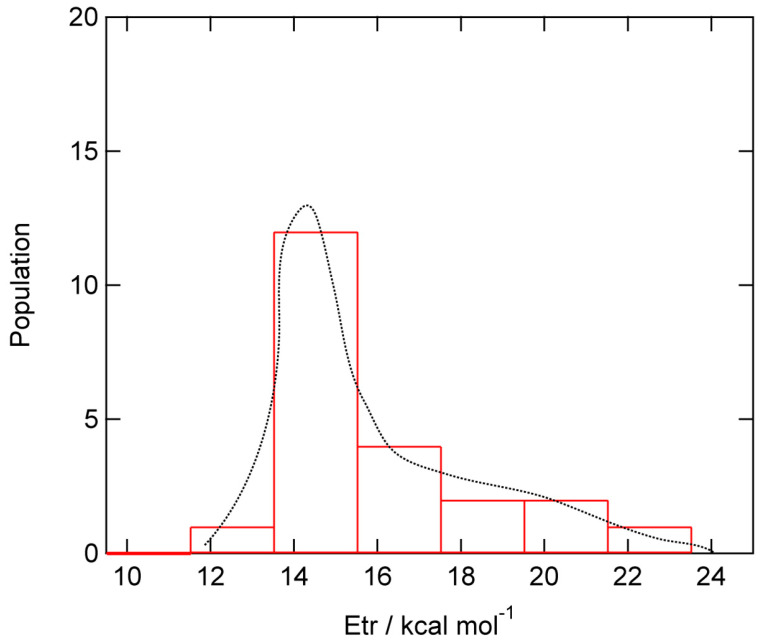
Distribution of translational energy of product H_2_ (Etr) formed by the H abstraction reaction for H + H-(GR)-H → H_2_ + (GR)-H. Direct AIMD calculation were performed at the CAM-B3LYP/6-31G(d) level.

A total of 22 trajectories were run at a collision energy of E_coll_ = 1.0 kcal/mol. The translational energies of product H_2_ (E_trans_) formed by the H abstraction reaction (H-(GR)-H + H → H-(GR) + H_2_) are given in Figure 7. E_trans_ was distributed in the range of 12.0–22.0 kcal/mol with the peak of E_trans_ = 15.0 kcal/mol. The average of E_trans_ was 14.9 kcal/mol, which is 26% of the total available energy. The remaining energies were transferred into the vibrational modes of the H_2_-stretching mode and deformation modes of GR.

The time evolution of PE for selected five trajectories are given in Figure S2. Also, the results of the DFT-APFD functional are given in Figure S3 for comparison. Similar results were obtained in both methods.

### 3.5. Hydrogen Addition Reactions to GR Surface

**Figure 8:**
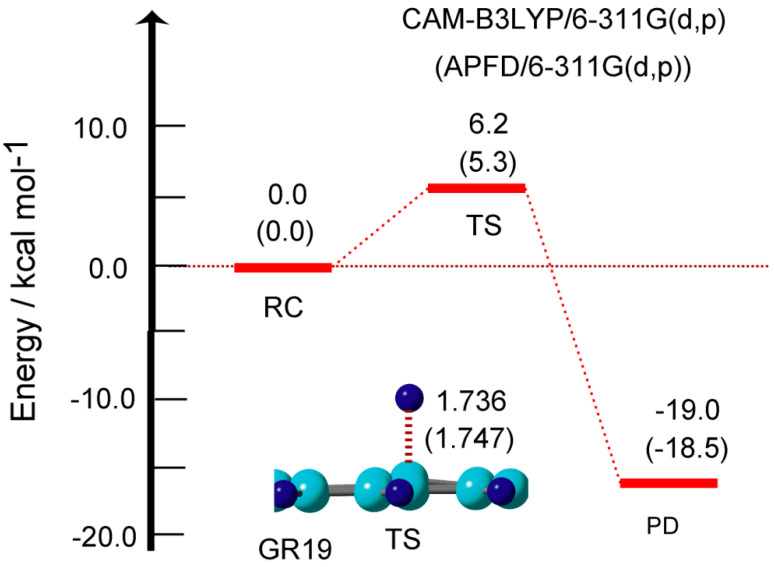
Energy diagram for the hydrogen addition reaction: GR + H at reactant state (RC: GR+H), and transition state (TS: H-GR-H), and product state (PD: GR-H+H). The relative energies and distances are in kcal/mol and Å, respectively. The calculations were carried out at the CAM-B3LYP/6-311G(d,p) level. The values obtained by APFD/6-311G(d,p) level are given in parenthesis. GR19 was used.

In previous sections, the hydrogen abstraction reaction was calculated and discussed. In this section, the hydrogen addition to the GR surface was re-examined. The reaction is expressed as GR + H → GR-H. In previous papers, we calculated the activation energies for the H atom addition reaction to the GR surface using the CAM-B3LYP functional. In this section, the energies were re-calculated using the APFD functional. The energy diagram and structure of TS for the H addition to the GR surface are given in Figure 8.

At TS, the H atom was located at 1.736 Å from the surface. In case of GR19, the activation energy was calculated to be Ea = 6.2 kcal/mol (CAM-B3LYP) and 5.3 kcal/mol (APFD). The calculated activation energies for several sized GRs are given in Table 1. The activation energies were 6.8 kcal/mol (GR04), 6.6 (GR07), and 5.6 (GR14), indicating that the activation energies were very low (Ea = 5–7 kcal/mol). This low activation barrier is due to the fact that the binding energies of H addition to the GR surface were large in the ranges 14.7–22.4 kcal/mol, as shown in Table 1. The similar results were obtained by the APFD functional. These results indicated that both the H addition and abstraction reactions proceed under low activation energy on GR.

Ea was largest in *n* = 14. This is due to the fact that the reaction energy (the binding energy of H to GR) was largest in *n* = 14. Namely, this reaction system is likely to be valid according to the Bell–Evans–Polanyi principle [35].

The potential energy curve for the second H atom addition reaction (GR-H + H → H-(GR)-H) is plotted in Figure S4. The atom approaches the carbon atom of GR-H spontaneously and binds to it. This reaction proceeds without activation barrier due to the radical–radical recombination between H atom and radical state of carbon atom.

### 3.6. Effects of DFT-Functional and Basis Sets on the Energetics

In the present calculation, the CAM-B3LYP/6-311G(d,p) level of theory was mainly used for the energy calculations. In this section, the effects of functionals on the energetics are investigated. The activation and binding energies calculated by APFD and wB97XD (including dispersion effects) are given in Table 1. The results obtained by APFD and wB97XD were in excellent agreement with those of the CAM-B3LYP functional. Therefore, there are no problems with the functions used. For the basis set, the calculations of def2-TZVP basis set were carried out for *n* = 4 system. The activation and binding energies were calculated to be 6.9 and 14.0 kcal/mol, respectively. These are also in excellent agreement with the others.

## 4. Discussion and Conclusions

### 4.1. Theoretical Design of H_2_ Storage/Release Device

**Figure 9:**
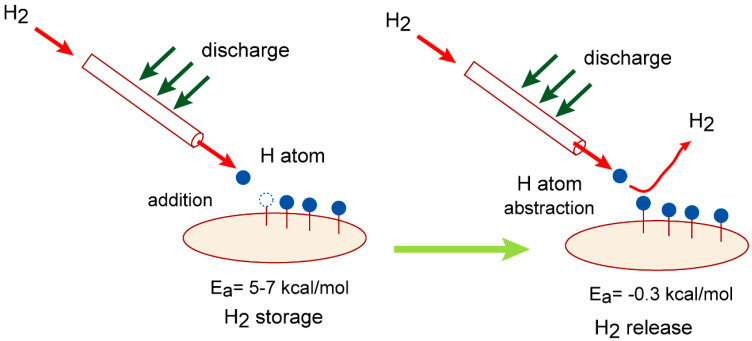
Schematic illustration of H_2_ storage/release device proposed in this study.

On the basis of the present calculations, the H_2_ storage and release device is designed in this section. A schematic illustration H_2_ storage/release device is given in Figure 9.

In case of a H_2_ storage process, first, the H_2_ gas is introduced into the reaction tube and H_2_ is discharged. The hydrogen atom (H atom) is easily generated: H_2_ + discharge → H + H. The H atom reacts with the carbon atom of the GR surface and GR-H and H-(GR)–H (hydrogenated GRs) are formed:

GR + H → GR–H

GR-H + H → H-(GR)–H

The first H atom addition reaction can proceed with a low activation energy (Ea = 5–7 kcal/mol), and the second addition reaction occurs without an energy barrier. The hydrogen molecule (H_2_) is chemically captured as H atoms on the GR surface. The present calculations indicated that the H_2_ storage process proceeds with low activation energy of 5–7 kcal/mol after the discharge.

In case of a H_2_ release process, the H atom is generated again by discharge of H_2_. The H atom reacts with the H atom on the GR surface and H_2_ is easily formed by the H abstraction reaction:

H + H-(GR)-H → H_2_ + H-(GR)

H + H-(GR) → H_2_ + GR

These reactions can also proceed without activation energy (Ea = −0.3 kcal/mol: negative activation energy). The present study thus designed a H_2_ storage/release device composed of graphene.

In order to proceed with the reaction efficiently, it would be best to move the reaction tube on the graphene substrate upon irradiation of the H atom (see Figure 9).

### 4.2. Scope and Limitation of the Present Study

In the present calculation, only one or two hydrogen atoms on the GR surface were examined. This means the reactions under a low-coverage condition. In order to confirm the present model in a large system, studying the effects of coverage on the reaction energies would be required. This extension will be carried out in the near future.

### 4.3. Conclusions

The development of hydrogen storage and release materials is one of the key themes in the development of a hydrogen society. In the present study, we theoretically designed hydrogen storage and release devices based on graphene (GR)—a lightweight and chemically stable material—using a direct AIMD approach. The calculations showed that the hydrogen addition reaction (H_2_ storage):

H + GR → (GR)-H

proceeds with low activation energy (5–7 kcal/mol). Also, the hydrogen abstraction from hydrogenated graphene (H_2_ release):

H + H-(GR)-H → H_2_ + H-(GR)

occurs without activation energy. The direct AIMD calculations indicate that H atom abstraction proceeds without the reaction barrier. The model designed in this study could be a new hydrogen storage/release device.

## Figures and Tables

**Table 1:** Activation energies for the hydrogen addition reaction (Ea in kcal/mol), GR(n) + H → GR(*n*)-H, calculated at the CAM-B3LYP, APFD or wB97XD/6-311G(d,p) levels. The binding energies of H atom to GR(*n*) are given in parenthesis (in kcal/mol).

*n*	CAM-B3LYP	APFD	wB97XD
4	6.8 (14.7)	6.6 (15.0)	8.8 (14.9)
7	6.6 (16.5)	5. 8 (16.4)	8.6 (16.9)
14	5.6 (22.4)	5.2 (20.9)	7.5 (23.6)
19	6.2 (19.0)	8.1 (19.8)	8.1 (19.8)

## Data Availability

The original contributions presented in this study are included in the article/Supplementary Materials. Further inquiries can be directed to the corresponding author(s).

## Supplementary Materials

The following supporting information can be downloaded at: https://www.mdpi.com/article/10.3390/nano15191498/s1, Figure S1: Intrinsic reaction coordinate (IRC) for the hydrogen abstraction reaction and the optimized structures along the IRC. The distances and bond lengths are in Å. The calculations were carried out at the CAM-B3LYP/6-311G(d,p) level; Figure S2: Time evolution of (A) snapshots and (B) potential energy of reaction system for hydrogen abstraction reaction, H + H-(GR)-H → H_2_ + GR-H. The results of 5 trajectories are plotted. Direct AIMD calculation were performed at the CAM-B3LYP/6-31G(d) level. The CAM-B3LYP/6-31G(d) -optimized geometry of H-(GR)-H was used as the initial geometry at time zero. The distances and bond lengths are in Å. GR04 was used; Figure S3: Time evolution of (A) snapshots and (B) potential energy of reaction system for hydrogen abstraction reaction, H + H-(GR)-H → H_2_ + GR-H. Direct AIMD calculation were performed at the APFD/6-31G(d) level. The APFD/6-31G(d)-optimized geometry of H-(GR)-H was used as the initial geometry at time zero. The distances and bond lengths are in Å. GR04 was used.; Figure S4: Energy profile for the second H atom addition reaction: H + H-(GR)-H → H_2_ + GR-H. The distances are in Å. The calculations were carried out at the CAM B3LYP/6-311G(d,p) level.
