# Supplementary material for: Molecular Design of H2 Storage/Release Devices: A Direct Ab Initio MD Study
Source: Nanomaterials (Basel). 2025 Oct 1;15(19):1498. doi: 10.3390/nano15191498 (PMC12525842; doi:10.3390/nano15191498)
Supplement: Supplementary file 1 [file nanomaterials-15-01498-s001.zip › nanomaterials-3818463-supplementary.pdf]

Supporting Information

Molecular Design of H_2 Storage/release Devices: A Direct ab initio MD Study.

Hiroto TACHIKAWA*

Division of Applied Chemistry, Faculty of Engineering

Hokkaido University, Sapporo 060-8628, JAPAN

Email: hiroto@eng.hokudai.ac.jp

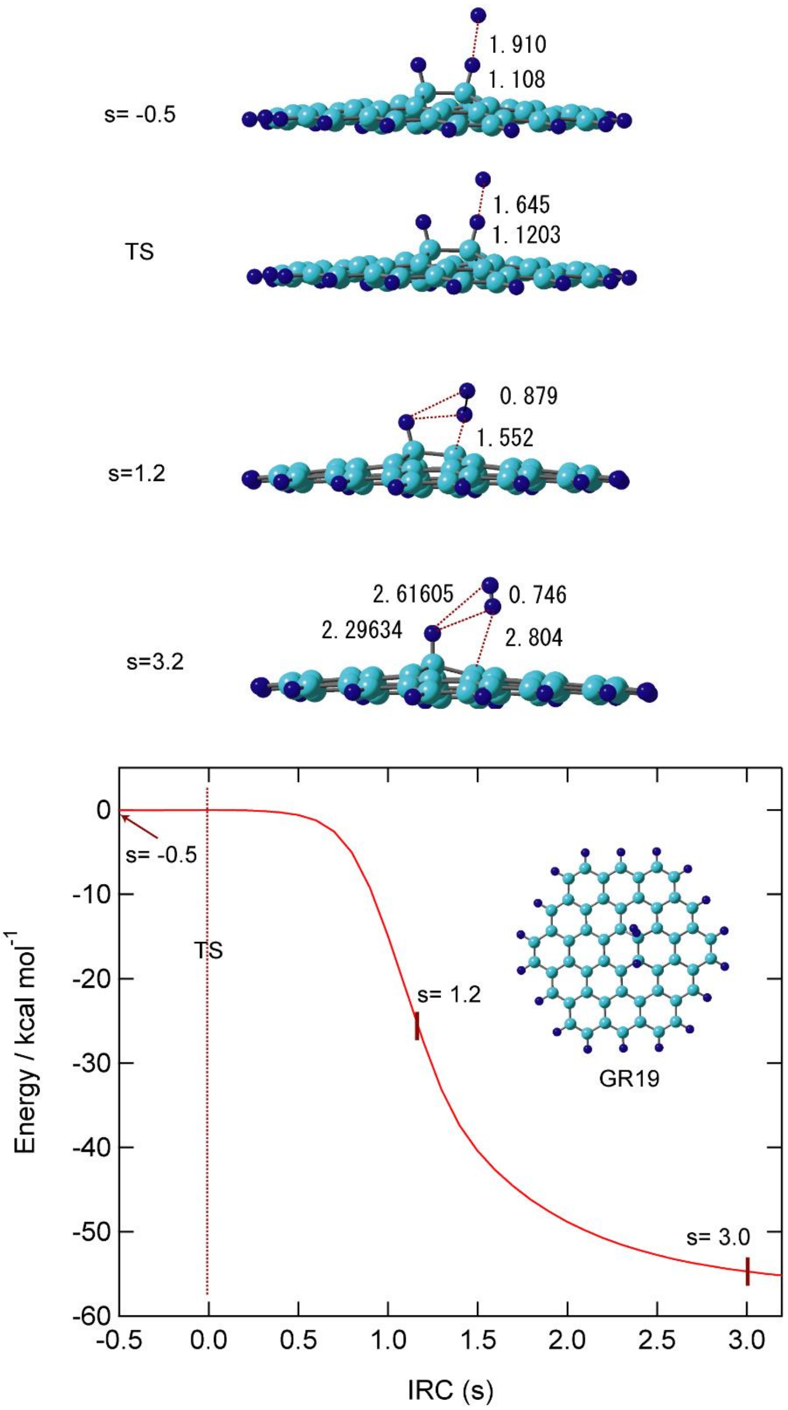

Figure S1. Intrinsic reaction coordinate (IRC) for the hydrogen abstraction reaction and the optimized structures along the IRC. The distances and bond lengths are in Å. The calculations were carried out at the CAM-B3LYP/6-311G(d,p) level.

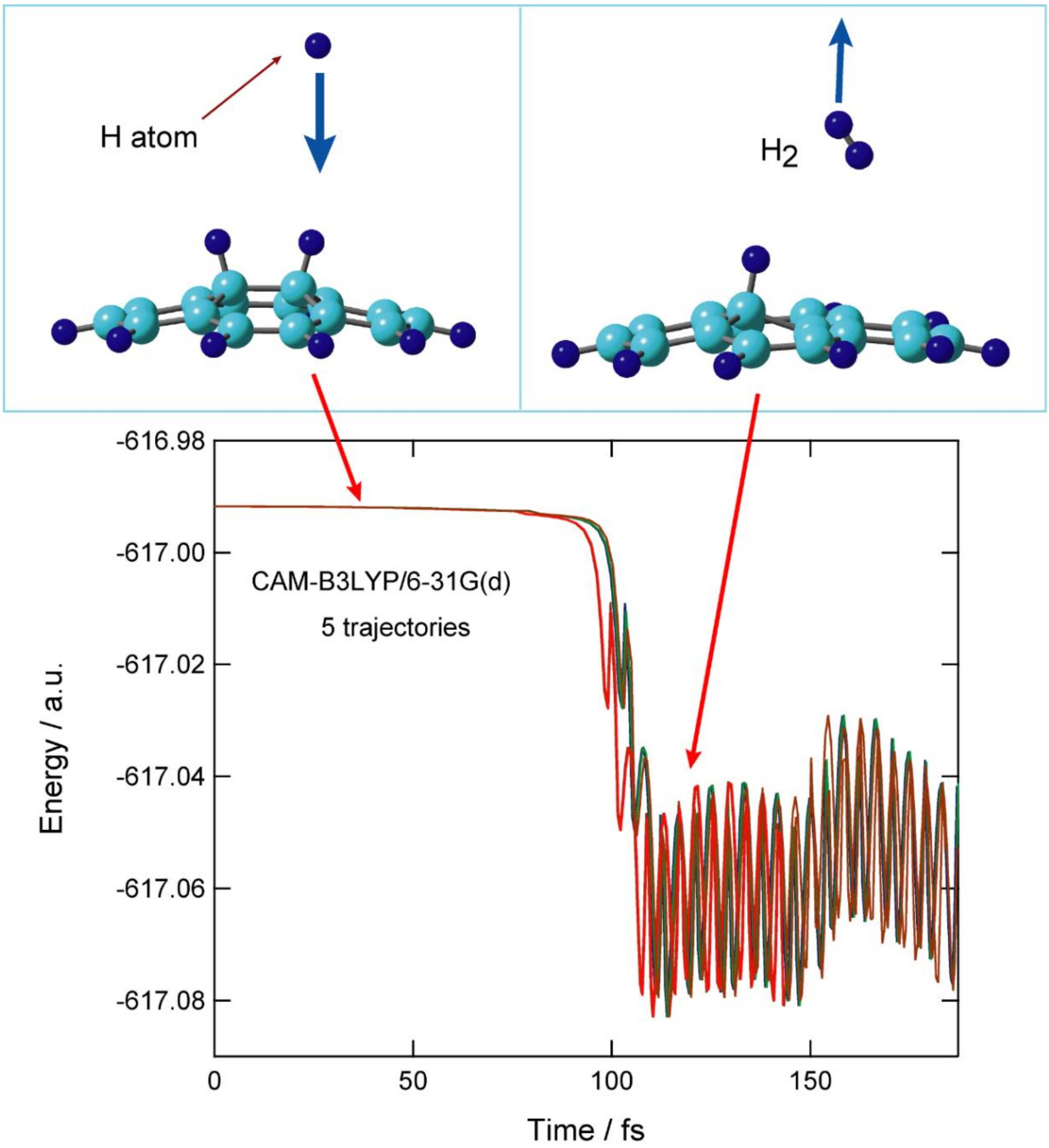

Figure S2. Time evolution of (A) snapshots and (B) potential energy of reaction system for hydrogen abstraction reaction, H + H-(GR)-H → H_2 + GR-H. Results of 5 trajectories are plotted. Direct AIMD calculation were performed at the CAM-B3LYP/6-31G(d) level. The CAM-B3LYP /6-31G(d)-optimized geometry of H-(GR)-H was used as the initial geometry at time zero. The distances and bond lengths are in Å. GR04 was used.

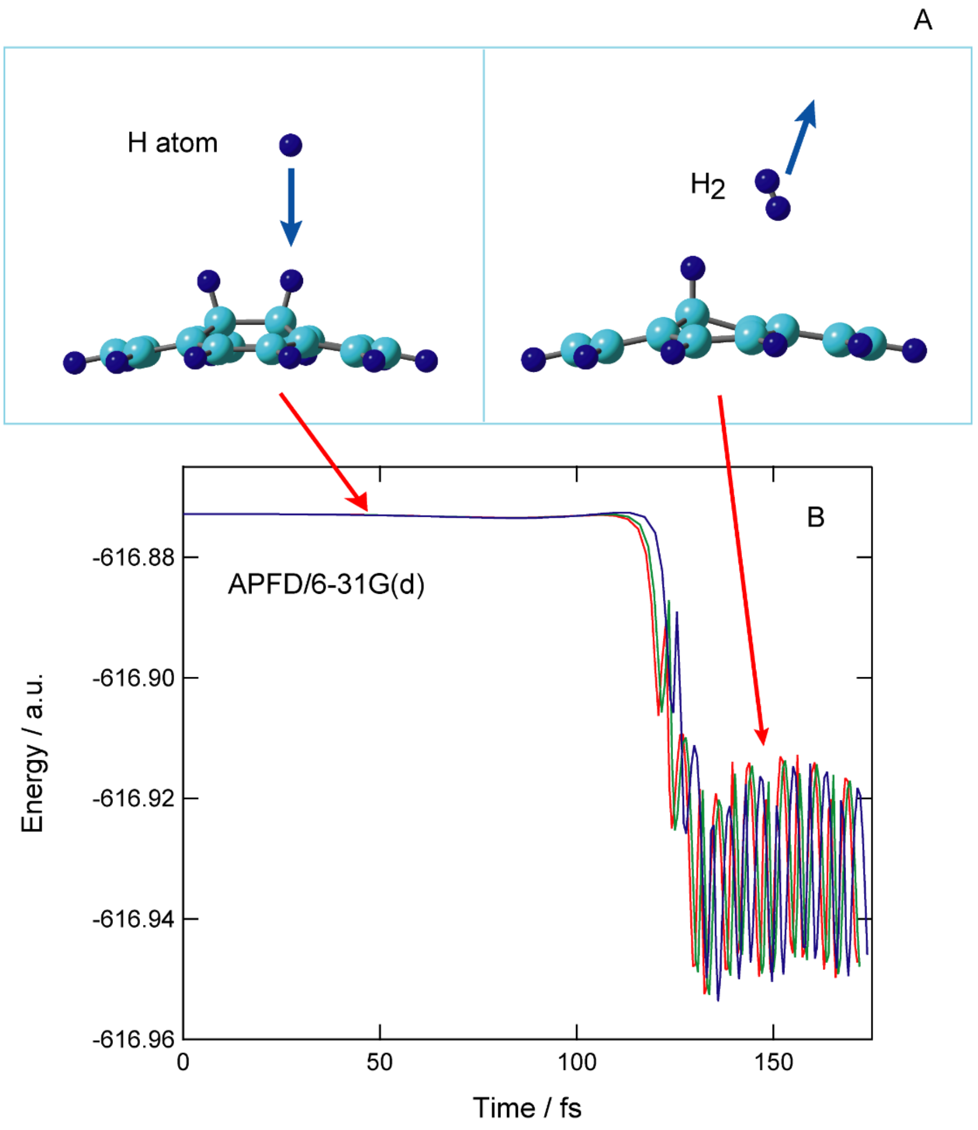

Figure S3. Time evolution of (A) snapshots and (B) potential energy of reaction system for hydrogen abstraction reaction, H + H-(GR)-H → H_2 + GR-H. Direct AIMD calculation were performed at the APFD/6-31G(d) level. The APFD/ 6-31G(d)-optimized geometry of H-(GR)-H was used as the initial geometry at time zero. The distances and bond lengths are in Å. GR04 was used.

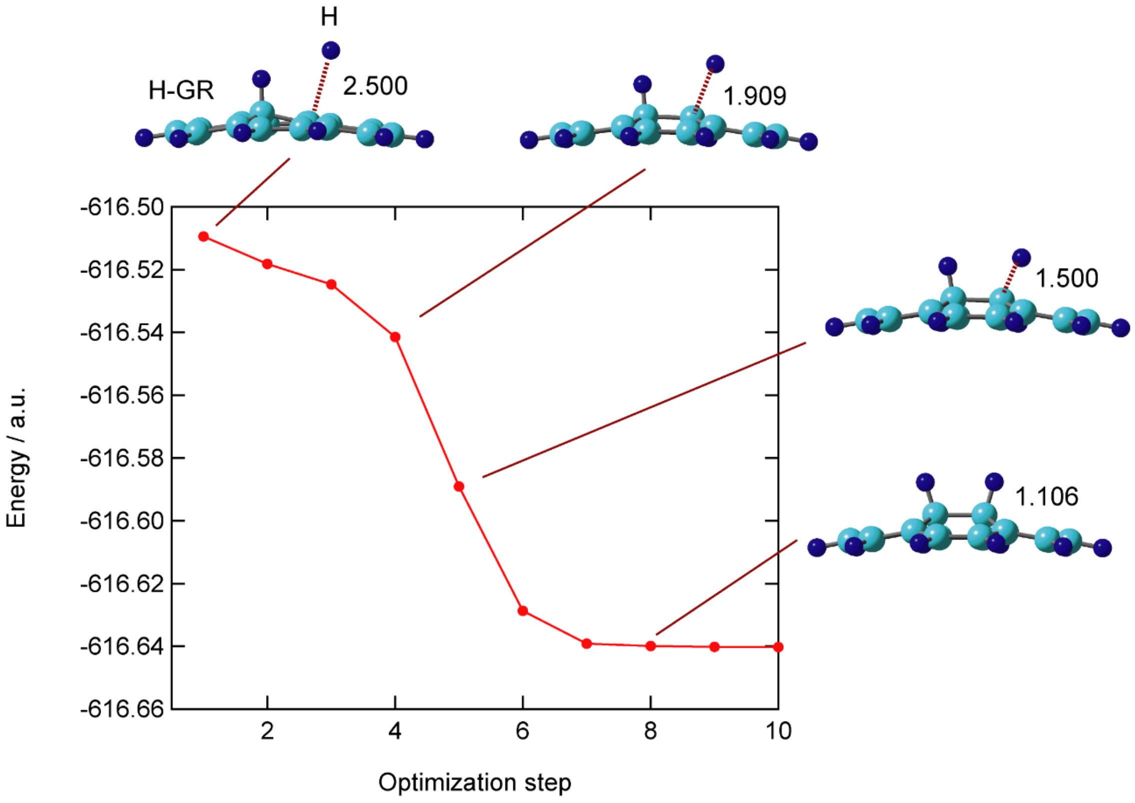

Figure S4. Energy profile for the second H-atom addition reaction: H-GR + H → H-(GR)-H. The distances are in Å. The calculations were carried out at the CAM-B3LYP/6-311G(d,p) level.

===== File ===

Cartesian coordinates of several optimized structures (in Å).

1. CAM-B3LYP/6-311G(d,p)-optimized structures of graphenes

GR04_graphene (Fig.S2)

C	0.00000000	0.00000000	3.50398600
C	0.00000000	1.20340300	2.81659400
C	0.00000000	1.22653300	1.42181700
C	0.00000000	0.00000000	0.71273000
C	0.00000000	-1.22653300	1.42181700
C	0.00000000	-1.20340300	2.81659400
C	0.00000000	2.45447400	0.67412700
C	0.00000000	0.00000000	-0.71273000
C	0.00000000	1.22653300	-1.42181700
C	0.00000000	2.45447400	-0.67412700
C	0.00000000	1.20340300	-2.81659400
H	0.00000000	2.14068400	-3.36130100
C	0.00000000	0.00000000	-3.50398600
C	0.00000000	-1.20340300	-2.81659400
C	0.00000000	-1.22653300	-1.42181700
C	0.00000000	-2.45447400	-0.67412700
C	0.00000000	-2.45447400	0.67412700
H	0.00000000	-3.38850300	1.22455000
H	0.00000000	-3.38850300	-1.22455000
H	0.00000000	3.38850300	1.22455000
H	0.00000000	0.00000000	4.58737000
H	0.00000000	2.14068400	3.36130100
H	0.00000000	-2.14068400	3.36130100
H	0.00000000	3.38850300	-1.22455000
H	0.00000000	0.00000000	-4.58737000
H	0.00000000	-2.14068400	-3.36130100

GR07_graphene (Fig.S2)

C	3.50778700	1.26584800	0.00009300
C	3.72847400	−0.07709600	0.00023600
C	2.64831900	−0.99906300	−0.00034900
C	1.33142400	−0.50225200	−0.00050900
C	1.10068100	0.90182400	−0.00050800
C	2.18943400	1.79383800	−0.00052800
C	0.23070700	−1.40411100	−0.00049200
C	−1.33142400	0.50225200	−0.00040700
C	−1.10068100	−0.90182400	−0.00046000
C	−2.18943400	−1.79383800	−0.00042700
C	−3.50778700	−1.26584800	0.00029400
C	−3.72847400	0.07709600	0.00049100
C	−2.64831800	0.99906300	−0.00014300
C	−0.23070800	1.40411000	−0.00044000
C	1.93097000	3.19021300	0.00012900
C	0.65759500	3.67059700	0.00030900
C	−0.45890100	2.79291900	−0.00023700
C	−1.79746300	3.26729800	0.00059300
C	−2.85021700	2.40473600	0.00065600
C	2.85021700	−2.40473600	0.00039900
C	1.79746300	−3.26729800	0.00038500
C	0.45890100	−2.79291900	−0.00034200
C	−0.65759500	−3.67059700	0.00025300
C	−1.93097000	−3.19021300	0.00017500
H	4.34376200	1.95626100	0.00003200
H	4.74138100	−0.46384100	0.00030800
H	−4.34376200	−1.95626100	0.00027400
H	−4.74138100	0.46384100	0.00064600
H	2.77238500	3.87400900	0.00010000
H	0.47778400	4.73982400	0.00042300
H	−1.96901900	4.33788300	0.00075600
H	−3.86612700	2.78349900	0.00087900
H	3.86612700	−2.78349900	0.00054100
H	1.96901900	−4.33788200	0.00050500

H	−0.47778400	−4.73982400	0.00032900
H	−2.77238500	−3.87400800	0.00018900

GR14_graphene (Fig.S2)

C	1.23261296	-0.00000000	2.82737800
C	0.00000000	0.00000000	3.53972800
C	-1.23261296	-0.00000000	2.82737800
C	-1.23332404	0.00000000	1.41239500
C	0.00000000	0.00000000	0.69911500
C	1.23332404	0.00000000	1.41239500
C	-2.45249104	0.00000000	0.70906300
C	-1.23332404	-0.00000000	-1.41239500
C	-2.45249104	0.00000000	-0.70906300
C	-3.68534504	0.00000000	-1.42900300
C	-3.66556904	0.00000000	-2.80831300
C	-2.45911104	0.00000000	-3.53207400
C	-1.23261296	-0.00000000	-2.82737800
C	-0.00000000	0.00000000	-0.69911500
C	2.45249104	0.00000000	0.70906300
C	2.45249104	0.00000000	-0.70906300
C	1.23332404	0.00000000	-1.41239500
C	1.23261296	-0.00000000	-2.82737800
C	-0.00000000	0.00000000	-3.53972800
C	-2.45911104	0.00000000	3.53207400
C	-3.66556904	0.00000000	2.80831300
C	-3.68534504	0.00000000	1.42900300
C	-4.91462504	0.00000000	0.67275000
C	-4.91462504	0.00000000	-0.67275000
C	2.45911104	0.00000000	3.53207400
C	0.00000000	0.00000000	4.94528000
H	-4.60342204	0.00000000	-3.35375700
C	-2.42470504	0.00000000	-4.95552600
C	3.68534504	0.00000000	1.42900300
C	3.68534504	0.00000000	-1.42900300

C	2.45911104	0.00000000	-3.53207400
C	-0.00000000	0.00000000	-4.94528000
C	-2.42470504	0.00000000	4.95552600
H	-4.60342204	0.00000000	3.35375700
H	-5.85001704	0.00000000	1.22092300
H	-5.85001704	0.00000000	-1.22092300
C	4.91462504	0.00000000	-0.67275000
C	4.91462504	0.00000000	0.67275000
C	3.66556904	0.00000000	2.80831300
C	3.66556904	0.00000000	-2.80831300
C	2.42470504	0.00000000	-4.95552600
C	1.24522896	-0.00000000	-5.63142600
C	-1.24522896	-0.00000000	-5.63142600
C	2.42470504	0.00000000	4.95552600
C	1.24522896	0.00000000	5.63142600
C	-1.24522896	-0.00000000	5.63142600
H	-3.36416104	0.00000000	-5.49657800
H	-3.36416104	0.00000000	5.49657800
H	5.85001704	0.00000000	-1.22092300
H	5.85001704	0.00000000	1.22092300
H	4.60342204	0.00000000	3.35375700
H	4.60342204	0.00000000	-3.35375700
H	3.36416104	0.00000000	-5.49657800
H	1.23884396	-0.00000000	-6.71568500
H	-1.23884396	-0.00000000	-6.71568500
H	3.36416104	0.00000000	5.49657800
H	1.23884396	0.00000000	6.71568500
H	-1.23884396	-0.00000000	6.71568500

GR19_graphene (Fig.S2)

C	-0.71213104	3.67706957	0.00000000

C	0.71213104	3.67706957	-0.00000000
C	1.41807299	2.45617447	0.00000000
C	0.70475649	1.22067405	-0.00000000
C	-0.70475649	1.22067405	-0.00000000
C	-1.41807299	2.45617447	0.00000000
C	1.40951298	0.00000000	-0.00000000
C	-0.70475649	-1.22067405	-0.00000000
C	0.70475649	-1.22067405	-0.00000000
C	1.41807299	-2.45617447	0.00000000
C	0.71213104	-3.67706957	0.00000000
C	-0.71213104	-3.67706957	0.00000000
C	-1.41807299	-2.45617447	0.00000000
C	-1.40951298	-0.00000000	-0.00000000
C	-2.82837014	2.45525836	0.00000000
C	-3.54050118	1.22181121	0.00000000
C	-2.83614598	-0.00000000	0.00000000
C	-3.54050118	-1.22181121	0.00000000
C	-2.82837014	-2.45525836	0.00000000
C	2.82837014	2.45525836	0.00000000
C	3.54050118	1.22181121	0.00000000
C	2.83614598	0.00000000	0.00000000
C	3.54050118	-1.22181121	0.00000000
C	2.82837014	-2.45525836	0.00000000
C	-1.42576745	4.90309564	0.00000000
C	1.42576745	4.90309564	-0.00000000
C	1.42576745	-4.90309564	0.00000000
C	-1.42576745	-4.90309564	0.00000000
C	-3.53332166	3.68629866	0.00000000
C	-4.95908911	1.21679699	0.00000000
C	-4.95908911	-1.21679699	0.00000000
C	-3.53332166	-3.68629866	0.00000000
C	3.53332166	3.68629866	0.00000000
C	4.95908911	1.21679699	0.00000000
C	4.95908911	-1.21679699	0.00000000
C	3.53332166	-3.68629866	0.00000000
C	-5.64451377	2.47931980	0.00000000

C	-4.96941082	3.64863242	-0.00000000
C	-2.81826322	4.88137508	0.00000000
C	-5.63652644	0.00000000	0.00000000
C	-5.64451377	-2.47931980	0.00000000
C	-4.96941082	-3.64863242	0.00000000
C	-2.81826322	-4.88137508	0.00000000
C	-0.67510295	6.12795222	0.00000000
C	0.67510295	6.12795222	-0.00000000
C	2.81826322	4.88137508	0.00000000
C	-0.67510295	-6.12795222	0.00000000
C	4.96941082	3.64863242	0.00000000
H	-6.72868531	2.47391053	0.00000000
H	-5.50681202	4.59025715	-0.00000000
H	-3.36074784	5.82098600	-0.00000000
H	-6.72149567	0.00000000	-0.00000000
H	-6.72868531	-2.47391053	0.00000000
H	-5.50681202	-4.59025715	0.00000000
H	-3.36074784	-5.82098600	-0.00000000
H	-1.22187329	7.06416768	0.00000000
H	1.22187329	7.06416768	-0.00000000
H	3.36074784	5.82098600	-0.00000000
C	4.96941082	-3.64863242	0.00000000
C	5.64451377	-2.47931980	0.00000000
C	5.63652644	-0.00000000	0.00000000
C	5.64451377	2.47931980	-0.00000000
C	2.81826322	-4.88137508	0.00000000
C	0.67510295	-6.12795222	0.00000000
H	-1.22187329	-7.06416768	0.00000000
H	5.50681202	4.59025715	0.00000000
H	5.50681202	-4.59025715	0.00000000
H	6.72868531	-2.47391053	0.00000000
H	6.72149567	0.00000000	-0.00000000
H	6.72868531	2.47391053	-0.00000000
H	3.36074784	-5.82098600	-0.00000000
H	1.22187329	-7.06416768	0.00000000

2. CAM-B3LYP/6-311G(d,p)-optimized structures (Figure 3)

Reactant (Fig.S3)

C	-5.63840600	0.01699500	-0.00869900
C	-4.96994900	1.22345700	-0.00080600
C	-0.71508300	3.65928800	-0.04484200
C	0.71508500	3.65928800	-0.04484200
C	4.96995000	1.22345500	-0.00080000
C	5.63840600	0.01699200	-0.00869200
C	-5.63892600	-2.45836000	-0.11392600
C	-2.83231800	2.45204200	0.03117200
C	2.83231900	2.45204100	0.03117500
C	5.63892500	-2.45836200	-0.11391900
C	-4.96420900	-3.62219300	-0.21169700
C	-4.95004400	-1.20738000	0.04358200
C	-3.54688900	1.22996600	0.09306500
C	-1.42745200	2.44179200	0.07459500
C	1.42745200	2.44179100	0.07459600
C	3.54688900	1.22996400	0.09306900
C	4.95004300	-1.20738100	0.04358800
C	4.96420800	-3.62219400	-0.21169200
C	-0.67254300	-6.05872800	-0.45959700
C	-2.81154800	-4.84270200	-0.28237500
C	-3.53203000	-3.66159100	-0.12463700
C	-3.55218200	-1.21366300	0.18904600
C	-2.84535700	0.01821800	0.24059000
C	-0.73031500	1.20429300	0.26047300
C	0.73031500	1.20429300	0.26047400
C	2.84535700	0.01821700	0.24059400
C	3.55218100	-1.21366400	0.18905000
C	3.53202800	-3.66159200	-0.12463300
C	2.81154700	-4.84270300	-0.28237200
C	0.67254100	-6.05872800	-0.45959600
C	-1.42536500	-4.85361700	-0.22462800
C	-2.83076300	-2.45734700	0.13431400

C	-1.43328600	0.03458500	0.39066700
C	1.43328500	0.03458500	0.39066900
C	2.83076200	-2.45734800	0.13431700
C	1.42536300	-4.85361700	-0.22462700
C	-0.72706900	-3.65024200	0.07011200
C	-1.43916500	-2.48029800	0.29111900
C	-0.77711700	-1.24389900	0.85367200
C	0.77711500	-1.24389900	0.85367300
C	1.43916300	-2.48029800	0.29112100
C	0.72706800	-3.65024300	0.07011300
H	1.06109500	-1.28483100	1.91422300
H	-1.06109700	-1.28483000	1.91422200
C	-3.53680900	3.68730600	-0.10594400
C	-1.41871400	4.87480200	-0.17608700
C	1.41871600	4.87480200	-0.17608600
C	3.53681000	3.68730500	-0.10594100
C	0.67639900	6.09134700	-0.29243600
C	-0.67639600	6.09134700	-0.29243700
C	-2.82413700	4.86273400	-0.19769400
C	2.82413900	4.86273300	-0.19769100
C	4.97763200	3.65077900	-0.15725200
C	5.65396300	2.48870700	-0.10976200
C	-4.97763000	3.65078100	-0.15725700
C	-5.65396200	2.48870900	-0.10976800
H	1.22456500	7.02197900	-0.38621600
H	-1.22456200	7.02198000	-0.38621600
H	-3.35537200	5.80289800	-0.30177900
H	3.35537500	5.80289700	-0.30177700
H	5.50816900	4.59200400	-0.24708200
H	6.73685700	2.48019600	-0.16150500
H	-5.50816700	4.59200600	-0.24708600
H	-6.73685600	2.48019900	-0.16151200
H	-6.71947400	0.00734600	-0.09916200
H	-6.72015800	-2.44219400	-0.19155200
H	-5.49501000	-4.55454400	-0.36762000
H	-3.34308900	-5.76818400	-0.47652900

H	-1.22246800	-6.97236400	-0.65511000
H	1.22246600	-6.97236400	-0.65511000
H	3.34308700	-5.76818400	-0.47652700
H	5.49500900	-4.55454600	-0.36761300
H	6.72015800	-2.44219700	-0.19154300
H	6.71947400	0.00734300	-0.09915400

TS (Fig.S3)

C	0.8189	-1.21076	0.82464
H	1.10834	-1.24274	1.89671
H	1.34149	-1.31957	3.6513
C	-5.63887	-0.18668	-0.01348
C	-5.01541	1.04373	-0.00525
C	-0.85345	3.6351	-0.04961
C	0.57561	3.68763	-0.05042
C	4.91756	1.40945	-0.0151
C	5.63018	0.2282	-0.02572
C	-5.54859	-2.66055	-0.11931
C	-2.92464	2.35034	0.02589
C	2.73617	2.55861	0.02094
C	5.72183	-2.24555	-0.13354
C	-4.83185	-3.79896	-0.2177
C	-4.90591	-1.38504	0.03685
C	-3.59351	1.10255	0.08657
C	-1.52034	2.39199	0.06766
C	1.33243	2.49681	0.06533
C	3.49536	1.36351	0.07877
C	4.98767	-1.02065	0.0244
C	5.09027	-3.43348	-0.23098
C	-0.45357	-6.07681	-0.47092
C	-2.63589	-4.93997	-0.29143
C	-3.39899	-3.78596	-0.13316
C	-3.50852	-1.34004	0.17967

C	-2.84761	-0.08288	0.23111
C	-0.77776	1.1808	0.24961
C	0.68134	1.23442	0.24748
C	2.83903	0.12624	0.22216
C	3.5906	-1.07846	0.16894
C	3.66054	-3.52553	-0.14377
C	2.98382	-4.73246	-0.29928
C	0.89069	-6.02713	-0.4725
C	-1.25004	-4.90007	-0.23633
C	-2.74201	-2.55648	0.12362
C	-1.43701	-0.0145	0.37817
C	1.4273	0.0899	0.37137
C	2.91563	-2.34736	0.11326
C	1.59874	-4.79473	-0.24011
C	-0.59596	-3.67151	0.05554
C	-1.35031	-2.52828	0.27686
C	-0.73188	-1.2683	0.83588
C	1.52562	-2.42102	0.26953
C	0.85683	-3.61776	0.05299
H	-1.00674	-1.32018	1.8987
C	-3.67433	3.55903	-0.10814
C	-1.60157	4.82417	-0.17769
C	1.23405	4.92853	-0.17913
C	3.39456	3.81917	-0.11319
C	0.44736	6.11722	-0.29228
C	-0.90455	6.06746	-0.29167
C	-3.00556	4.76026	-0.19821
C	2.63888	4.96797	-0.20144
C	4.83565	3.83577	-0.16524
C	5.55441	2.69919	-0.12099
C	-5.1128	3.46944	-0.15792
C	-5.74575	2.28306	-0.1115
H	0.96084	7.06761	-0.38414
H	-1.48656	6.97755	-0.38294
H	-3.57119	5.68041	-0.29974
H	3.13526	5.92724	-0.30333

H	5.33126	4.79606	-0.25275
H	6.63686	2.7308	-0.17301
H	-5.67777	4.39061	-0.24548
H	-6.82765	2.23479	-0.16189
H	-6.719	-0.23609	-0.1021
H	-6.62982	-2.68414	-0.19504
H	-5.32855	-4.75014	-0.37226
H	-3.13357	-5.88445	-0.48382
H	-0.96958	-7.01041	-0.66457
H	1.47368	-6.92016	-0.6673
H	3.54895	-5.63804	-0.49243
H	5.65501	-4.34575	-0.38632
H	6.80175	-2.1899	-0.21101
H	6.71086	0.25875	-0.116

--

Product (Fig.S3)

C	-4.88850200	-2.79686800	-0.06515900
C	-4.91414200	-1.41031300	-0.04835200
C	-2.45451500	2.83711500	0.01345000
C	-1.21920700	3.54229600	0.03193100
C	3.69498900	3.53037300	-0.05813600
C	4.89399700	2.81221100	-0.09624100
C	-3.65373500	-4.94844900	-0.12519100
C	-3.68425500	0.72433500	0.01205300
C	1.23292400	3.53754600	0.03193200
C	6.13786400	0.66936600	-0.12810100
C	-2.48973400	-5.62980700	-0.16768400
C	-3.68284500	-3.51433500	-0.01782900
C	-3.68753100	-0.69441100	0.03204400
C	-2.46300200	1.42524500	0.05717800
C	0.00548600	2.82799000	0.08497700
C	2.46549100	2.82760200	0.01345100
C	4.91642500	1.42455200	-0.06910400

C	6.13452200	-0.68055900	-0.12132600
C	2.46791100	-5.63939700	-0.16761400
C	-0.01090300	-5.63382800	-0.14954500
C	-1.22435900	-4.95446400	-0.08578500
C	-2.46508400	-2.82054400	0.09926400
C	-2.46664200	-1.39819500	0.13669400
C	-1.23556400	0.70510900	0.16473300
C	0.00275000	1.41801500	0.17066100
C	2.46849300	1.41572800	0.05716900
C	3.68704300	0.71009400	0.01204700
C	4.90863200	-1.42927700	-0.04832300
C	4.87760900	-2.81575900	-0.06511200
C	3.63452400	-4.96255400	-0.12510800
C	1.20513000	-4.95917100	-0.08576600
C	-1.22051100	-3.54329500	0.08016500
C	-1.25740300	-0.68227300	0.24688600
C	1.23827300	0.70034700	0.16471300
C	3.68481300	-0.70865100	0.03204500
C	3.66920400	-3.52853300	-0.01777800
C	1.20675400	-3.54796900	0.08017400
C	-0.00555100	-2.86855600	0.19903300
C	-0.00276000	-1.42115500	0.59728400
C	1.25473700	-0.68713300	0.24686500
C	2.46119100	-1.40771600	0.13668900
C	2.45413200	-2.83003400	0.09928700
H	-0.00280300	-1.44969800	1.71058000
C	-4.91087200	1.44356300	-0.06910900
C	-3.68125300	3.54464000	-0.05814500
C	-1.20947500	4.95829800	-0.02689100
C	1.22868400	4.95360000	-0.02688500
C	-2.46553700	5.64736500	-0.08104200
C	-3.63965500	4.97606500	-0.09651200
C	-4.88306500	2.83109200	-0.09625300
C	0.01094300	5.63572800	-0.04368700
C	2.48744300	5.63779500	-0.08102500
C	3.65893600	4.96196600	-0.09648900

C	-6.13524100	0.69307800	-0.12811900
C	-6.13713400	-0.65683300	-0.12135300
H	-2.45489900	6.73088000	-0.11642400
H	-4.57808500	5.51693200	-0.14365700
H	-5.81791000	3.37846200	-0.15604400
H	0.01302600	6.71955400	-0.08759900
H	2.48099800	6.72134300	-0.11640000
H	4.59946500	5.49917500	-0.14362000
H	-7.06908800	1.24088700	-0.18566100
H	-7.07221700	-1.20293700	-0.17428300
H	-5.82223100	-3.34390500	-0.14086300
H	-4.59862300	-5.47547100	-0.19473700
H	-2.48978300	-6.70914100	-0.26966300
H	-0.01301500	-6.71315900	-0.25908300
H	2.46376300	-6.71872600	-0.26957400
H	4.57737000	-5.49322700	-0.19461700
H	5.80921900	-3.36639500	-0.14081400
H	7.06749000	-1.23026400	-0.17425000
H	7.07383500	1.21354100	-0.18563300
H	5.83097400	3.35592900	-0.15601000
